# Colloidal Nanoparticles Isolated from Duck Soup Exhibit Antioxidant Effect on Macrophages and Enterocytes

**DOI:** 10.3390/foods12050981

**Published:** 2023-02-25

**Authors:** Ligen Xu, Mingcai Duan, Zhaoxia Cai, Tao Zeng, Yangying Sun, Shuang Cheng, Qiang Xia, Changyu Zhou, Jun He, Lizhi Lu, Daodong Pan

**Affiliations:** 1 Key Laboratory of Animal Protein Food Deep Processing Technology of Zhejiang Province, College of Food and Pharmaceutical Sciences, Ningbo University, Ningbo 315832, China; 2 Institute of Animal Husbandry and Veterinary Science, Zhejiang Academy of Agricultural Sciences, Hangzhou 310021, China; 3 Hubei Hongshan Laboratory, National Research and Development Center for Egg Processing, College of Food Science and Technology, Huazhong Agricultural University, Wuhan 430070, China; 4 Zhejiang-Malaysia Joint Research Laboratory for Agricultural Product Processing and Nutrition, Ningbo University, Ningbo 315832, China

**Keywords:** duck soup, colloidal nanoparticles, RAW 264.7, Caco-2, antioxidant effect

## Abstract

Food-derived colloidal nanoparticles (CNPs) have been found in many food cooking processes, and their specific effects on human health need to be further explored. Here, we report on the successful isolation of CNPs from duck soup. The hydrodynamic diameters of the obtained CNPs were 255.23 ± 12.77 nm, which comprised lipids (51.2%), protein (30.8%), and carbohydrates (7.9%). As indicated by the tests of free radical scavenging and ferric reducing capacities, the CNPs possessed remarkable antioxidant activity. Macrophages and enterocytes are essential for intestinal homeostasis. Therefore, RAW 264.7 and Caco-2 were applied to establish an oxidative stress model to investigate the antioxidant characteristics of the CNPs. The results showed that the CNPs from duck soup could be engulfed by these two cell lines, and could significantly alleviate 2,2′-Azobis(2-methylpropionamidine) dihydrochloride (AAPH)-induced oxidative damage. It indicates that the intake of duck soup is beneficial for intestinal health. These data contribute to revealing the underlying functional mechanism of Chinese traditional duck soup and the development of food-derived functional components.

## 1. Introduction

Food-grade nanoparticles, such as nanoemulsions and liposomes, have been successfully developed with excellent stability and efficacy [1,2]. Soups can produce colloidal nanoparticles (CNPs) in the case of self-assembly, which have been observed in soups made with clams [3], Chinese medicine [4], and pig bones [5]. Nutrients migrate from food raw materials to water, and then form self-assembling particles between molecules that interact with each other covalently and noncovalently during heating, ranging in size from the nanometer to the micron scale. Soups have rich flavors and complex components, and the CNPs in them change the degree of digestion and the absorption of nutrients in raw materials [6,7], whereas further research is needed to investigate the ingestion and functioning of these nanoparticles.

Macrophages are immune cells that serve a variety of purposes. They are widely distributed throughout the body and serve as significant study subjects for cellular phagocytosis, cellular immunity, and molecular immunology. Macrophages have a strategic role in intestinal homeostasis and intestinal physiology [8]. RAW 264.7 is considered to be one of the best models of macrophages, and it has several uses in the study of inflammation, immunity, apoptosis, and tumor research [9,10]. The colonic mucosal epithelium is the fulcrum that maintains intestinal homeostasis, and these barrier-forming cells can precisely control redox signaling and thus avoid tissue damage [11]. The interactions between food and intestine have been studied using the Caco-2 cell model, which is well adapted for this purpose [12].

Oxidative stress is caused by the imbalance between oxidation and antioxidation in biological systems. It usually leads to the excess accumulation of reactive oxygen species (ROS) and induces the damage of cellular components and cell apoptosis [13,14]. A large number of studies on oxidative stress and anti-inflammatory processes have conducted out in Caco-2 cells or RAW 264.7 cells, confirming the representativeness of such cell models [15,16,17]. Therefore, studying the interactions of food-derived CNPs with both macrophages and enterocytes should be appropriate for revealing the effects of CNPs on intestinal health.

Duck meat, as a quality and nutritious meat resource, is becoming more and more well-liked by consumers worldwide, especially in Asia [18]. In China, old duck is often used as the raw material for duck soup, and is it believed to have a curative effect on inflammation. The aged ducks (500 days of age) were found to have a significant antioxidant capacity, with abundant metabolites [19]. However, the CNPs in duck soup, and their biological effects on intestine have not been characterized yet.

To further understand the bioactivity of duck soup, the CNPs were isolated from duck soup, and their interactions with Caco-2 cells and RAW 264.7 cells were investigated to reveal the antioxidant effects of these CNPs on the intestinal tract. Therefore, this work should expand our knowledge of the biological function mechanism of food soup, and contribute to the development of gastrointestinal protection.

## 2. Materials and Methods

### 2.1. Materials

All the analytical-grade reagents used were purchased from Sinopharm Chemical Reagent Co., Ltd. (Shanghai, China), including sodium dihydrogen phosphate, glucose, sodium chloride, sodium hydroxide, and disodium hydrogen phosphate. Triglyceride kits, BCA protein assay kits, total antioxidant capacity colorimetric (T-AOC) assay kits (FRAP method), 2,2′-Azinobis-(3-ethylbenzthiazoline-6-sulphonate) (ABTS), cell counting kit-8 (CCK-8), and 1,1-diphenyl-2-trinitrophenylhydrazine (DPPH) were provided by Sangon Biotech Co., Ltd. (Shanghai, China). The following products were purchased from Sigma-Aldrich Co., Ltd. (Shanghai, China): Hoechst 33342 staining solution, dimethyl sulfoxide (DMSO), DiBAC4(3) staining solution, 2,2′-Azobis(2-methylpropionamidine) dihydrochloride (AAPH), and penicillin–streptomycin (100×, Sterile). The following products were obtained from Invitrogen Co., Ltd. (Carlsbad, CA, USA): Fetal bovine serum (FBS), 25% Pancreatin + Ethylene Diamine Tetraacetic Acid (EDTA), Dulbecco’s modified minimal essential medium (DMEM), MitoSOX Red staining solution, phosphate buffered saline (PBS), Hank’s balanced salt solution (HBSS), and minimal essential medium (MEM). The RAW 264.7 and Caco-2 cell lines were procured from the BeNa Culture Collection (Su Zhou, China). The HiPrep 16/60 Sephacryl S-500HR (1.0 × 120 cm) column was purchased from General Electric Company (Fairfield, CT, USA). Cell culture flasks, 96-well plates (black transparent flat bottom), and 24-well plates were purchased from Corning Company (Corning, NY, USA).

### 2.2. The Preparation of Duck Soup

Fresh 600-day-old Sheldrake carcasses (Huaying, Xinyang, China) were purchased. The duck breast meat was cut into square pieces with a side length of about 2 cm, blanched in boiling water to remove blood, and then washed on the surface in clean water. Meat pieces were cooked in deionized water (meat/water, *w/v*, 1:3) for 3 h at 100 °C, and heated with an induction cooker (300 W), during which the water loss caused by cooking was supplemented according to the liquid level. The duck soup was filtered twice with eight layers of cotton gauze to remove solids, and then stored at −40 °C for future use [6].

### 2.3. Separation of the CNPs from the Duck Soup

The CNPs of duck soup were separated according to a reported method, with some modifications [6]. The duck soup was filtered through a 0.45 μm filter membrane after being centrifuged at 400× *g* for 10 min. Four milliliters of duck soup supernatant were separated using a pre-equilibrated chromatographic column equipped with AKTA avant150 (General Electric Company, Fairfield, Connecticut). The concentration of phosphate buffer was adjusted to 0.02 M. The flow rate was 1 mL/min. The automatic collector was set at 4 mL/tube, and the UV monitor was set at 280 nm. The eluent at each stage isolated from the column was labeled as F_n_ (n for the scientific count), depending on the peak time.

Each F_n_ (1 mL) was gently injected into the sample pool of dynamic light scattering (DLS) (Malvern, UK) at 25 °C for measurement. The viscosity and refractive index (RI) were 0.8872 and 1.330, respectively [5]. The CNPs were selected from the eluents based on the polymer dispersity index (PDI), the hydrodynamic diameter, light scattering, and the ζ-potential. Each determination was repeated three times.

### 2.4. The Morphologies of the CNPs

The CNPs were dripped onto the copper net covered with Formvar films; the excess solution was slowly wiped off with filter paper along the edge of the copper mesh, and dripped with 1% uranyl acetate for dyeing. Morphologies were observed under a transmission electron microscope (TEM) at 80 kV.

### 2.5. Major Compositions Analysis of the CNPs

The major compositions (lipids, proteins, and carbohydrates) of the obtained CNPs were detected [20]. A protein detection was performed according to the method of BCA protein quantitative kits. An anthrone-sulfuric acid test was used to assess the number of polysaccharides in CNPs. According to the method provided by the kits, the content of triglyceride in the sample was measured using the GPO-PAP enzyme assay. The absorbance measurement was conducted with a multifunctional microplate reader (Infinite 200 PRO, TECAN, Switzerland). All of the above indexes were tested 3 times.

### 2.6. Determination of Antioxidant Activities

The ABTS free radical scavenging capacity of CNPs was evaluated based on a published method, with slight modifications [21]. The ABTS powder was weighed and prepared to 7 mM, and the potassium persulfate reagent was weighed and prepared to 140 mM. Then, 5 mL of ABTS solution was mixed with 88 μL potassium persulfate solution and placed away from light for 12–16 h. The mixture was then diluted 50 times, with distilled water as the ABTS+ reserve solution. The ABTS+ reserve solution (200 μL) and the sample solution (50 μL) were absorbed and added into the enzyme-labeled plate. The absorbance was measured at 734 nm after standing for 10 min in a dark environment at room temperature, and the measurement was repeated 3 times. The reported method was used to test the ferric reducing antioxidant power (FRAP) of the CNPs [20]. The 100 mM FeSO_4_·7H_2_O solution was diluted with deionized water to 0.15, 0.30, 0.60, 0.90, 1.20, and 1.50 mM as the standard for calibration curves. Samples (5 μL) and FeSO4·_7_H_2_O standard (5 μL) were added into 96-well plates in equal quantities, then FRAP solution reagent (5 μL) was added into each well and incubated at 37 °C for 5 min, and distilled water (5 μL) was used as the control. Finally, absorbance was measured at the wavelength of 593 nm, and test temperature was set at 37 °C, and the measurement was repeated 3 times. The DPPH radical scavenging ability of CNPs was tested, the published method was slightly modified (the ratio of DPPH solution to sample solution was 1:1, 100 µL) [22]. The 0.1 mM DPPH solution (100 μL) and sample solution (100 μL) were absorbed, then added to 96-well plates and mixed. After being kept in dark for 30 min, the plate was placed on the enzyme label analyzer, the absorbance was recorded at 517 nm, and the measurement was repeated 3 times.

### 2.7. Toxicity Test of the CNPs on Cells

Using the CCK-8 kit, the toxicity of CNPs to Raw 264.7 and Caco-2 cells was investigated. The cells (100 µL, 5 × 10^4^ cell/well) were inoculated into 96-well plates and incubated overnight in incubators (37 °C, 5% CO_2_). Each well of the cells was incubated for 24 h with 100 μL of CNPs at different concentrations, and mixed with 10 μL of CCK-8 solution for 4 h. The absorbance values were measured at 450 nm, and the cell viability was calculated by referring to Gao et al. [23]. Each determination was repeated three times.

### 2.8. Observation of the Uptake of CNPs by Raw 264.7 Cells and Caco-2 Cells

Nile red (1 µg/mL) was mixed with CNPs (1 mg/mL) and incubated for one hour at 40 °C. The filtrate was extracted via centrifugation at 4000× *g* for 5 min. The retained particles were washed with HBSS and centrifuged, and repeated several times until no red fluorescence was observed in the filtrate. The remaining particles were re-suspended in HBSS for use.

The cell suspension was inoculated at 5 × 10^4^ cell/well in 24-well plates, and incubated overnight (37 °C, 5% CO_2_). Then, the medium was removed and HBSS was used to wash the cells twice. The cells were fixed with 4% paraformaldehyde and stained with Hoechst 33342 (1–10 µg/mL).

The Hoechst 33342-stained cells and Nile Red-tagged colloidal particles were mixed and incubated for 3 h. Fluorescence was observed via inversed fluorescent microscope (IX-53, Olympus, Japan). The excitation and emission wavelengths of Nile red were 549 nm and 628 nm, respectively. The excitation and emission wavelengths of Hoechst 33342 were 346 and 460 nm, respectively. The instrument provided software for observation under a unified background, and the whole experiment was carried out three times in a dark environment [20].

### 2.9. Detection of Cell Membrane Potential and Mitochondrial Superoxide

DiBAC4 (3) staining solution (5 µm) and Mito-sox Red staining solution (2.5 µm) were applied for the determination of cell membrane potential and mitochondrial superoxide, respectively, using HBSS as the solvent. The procedures were as follows: 200 μL of 5 × 10^4^ cell/well cells were seeded in a black 96-microwell plate and cultured overnight in an incubator (37 °C, 5% CO_2_). The staining solution was added to each well at a dosage of 100 µL, and the excess staining solution was cleared after a certain period of incubation (30 min for DiBAC4 (3) and 10 min for Mito-Sox Red). Then, 100 µL of various concentrations (100 µg/mL, 500 µg/mL, and 1000 µg/mL) of the CNPs, and HBSS (control) were added, and then 50 µL AAPH (6.4 µm) was added and incubated for 30 min. Finally, 510 nm and 580 nm were chosen as the excitation and emission wavelengths, respectively, and the fluorescence intensity was observed under an inverted fluorescence microscope. Each determination was repeated three times.

### 2.10. Statistical Analysis

The data were presented as mean ± standard deviation. Statistical differences were examined via a one-way analysis of variance (ANOVA) combined with Duncan multiple comparison. The significance level was set at *p* < 0.05. Graphs were performed by Origin 2019 (Origin Lab, Northampton, MA, USA).

## 3. Results and Discussion

### 3.1. Isolation and Properties of the CNPs

**Figure 1:**
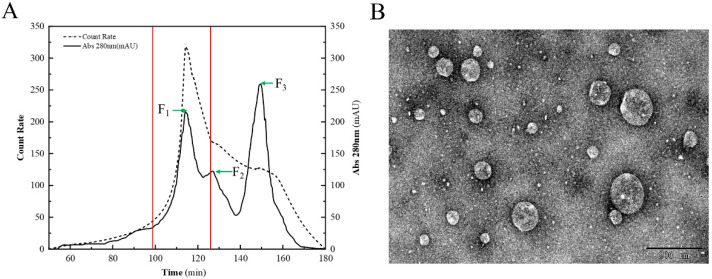
Chromatographic isolation and TEM of CNPs. (**A**) Isolation of the CNPs with a combination of chromatography and DLS. The solid line indicates the UV absorbance, and the dashed line indicates the light scattering intensity. F_1_, F_2_, and F_3_ are the three eluents collected at different periods, and are indicated by green arrows, and the range of F_1_ is marked by red lines. (**B**) TEM micrograph of the CNPs.

Three eluents, F_1_, F_2_, and F_3_, were separated and collected in the range of 100 to 160 min, among which F_1_ in the range of 100 to 120 min had a stronger signal of light scattering intensity than other eluents (Figure 1A). As shown in Table 1, the average hydrodynamic diameters of F_1_, F_2_, and F_3_ were 255 nm, 220 nm, and 147 nm, respectively, and the light scattering intensity in F_1_ was roughly three times greater than in F_2_. It has been indicated that larger particle sizes of CNPs are more efficiently phagocytosed by macrophages [24]. The minimum PDI of F_1_ indicated a narrow sample size distribution, while the particles in F_2_ and F_3_ may not have a uniform size. The maximum negative ζ-potential of F_3_ indicated a greater ionic bond interaction with the chromatographic gel, resulting in a delay in separation. The TEM micrograph of F_1_ confirmed that the particles contained in F_1_ had a uniform spheroid shape (Figure 1B). Thus, the representative F_1_ was chosen to learn the nano-functional properties of the CNPs from duck soup.

### 3.2. Major Components and Antioxidant Activities of CNPs

The CNPs obtained after 3 h of continuous simmering of the duck soup had a lipids content of 51.2%, followed by 30.8% of proteins and 7.9% of sugars (Table 2). It has been found that the constituent proteins in the particles are mainly associated with antioxidant activity [25], and that the second most abundant protein in CNPs offers the possibility of antioxidant activity. Protein extracts from duck meat have been shown to have a good ability in antioxidant and free radical scavenging [26]. The comparison revealed that the composition of CNPs in the duck soup was similar to that of porcine bone soup [20], but significantly different from that of freshwater clam [23], which contained 60% carbohydrates. This may be due to the differential compositions between clams and duck meat, indicating that the formation of CNPs in the soup should be closely associated with the ingredients of raw materials.

**Figure 2:**
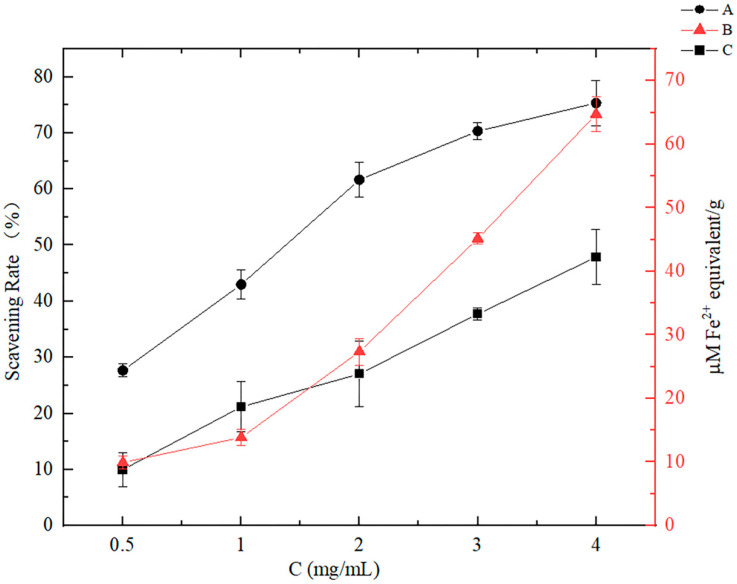
Antioxidant activities of the CNPs in vitro under different concentration conditions. A: Measured using ABTS method, expressed as free radical scavenging activity (%). B: Measured using FRAP analysis, expressed as Fe^2+^ equivalent. C: Measured using DPPH method, expressed as free radical scavenging activity (%).

It is common practice to utilize spectrophotometric techniques to assess food antioxidant potential, including the determinations of ABTS and DPPH, both of which involve the scavenging of free radicals [27]. Another method monitoring the iron ion reducing capacity is expressed as FRAP, and a high FRAP value indicates a stronger antioxidant activity [28]. As the CNP concentration increased, the antioxidant capacity showed an increasing trend in Figure 2, demonstrating that CNPs had a powerful antioxidant capability, but the effect of the high concentration of CNPs on cells needs to be explored.

### 3.3. Cytotoxicities of CNPs

Some nanoparticles that are used as food additives are toxic to Caco-2 cells, disrupting the cell tight junction permeability barrier and exacerbating the intestinal barrier injury inflammatory response caused by oxidative stress [29,30]. Similarly, it has been found that SiO_2_ CNPs have cytotoxic effects on macrophages at high concentrations [31]. However, self-assembled nanoparticles derived from porcine bone and freshwater clam had a protective effect on cells [23,32].

**Figure 3:**
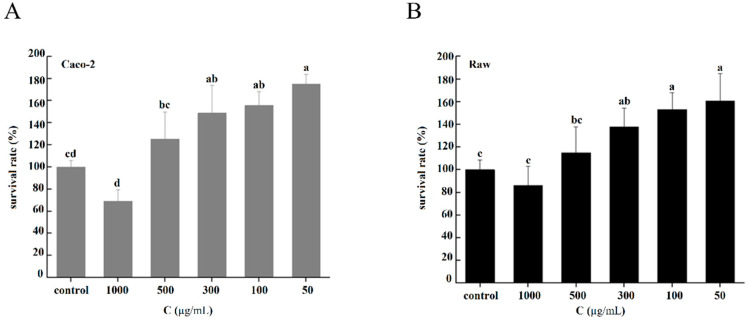
Cytotoxicities of the CNPs under different concentration conditions. (**A**) Cytotoxicities of CNPs on Caco-2 cells. (**B**) Cytotoxicities of CNPs on Raw 264.7 cells. Different superscript letters indicate significant differences between CNPs with different concentrations at the same time point (*p* < 0.05).

There was no significant difference in cell activity between the CNP treatments and the control, indicating that CNPs had no significant toxicity to Caco-2 cells (Figure 3A) or Raw 264.7 cells (Figure 3B), and could significantly promote the growth and proliferation of these cells when the concentration was 50–300 µg/mL. However, when the concentration of CNPs gradually increased; as can be seen from Figure 3, the cell activity showed a trend of decline.

### 3.4. Interactions of Caco-2 Cells and Raw 264.7 Cells with CNPs

Nile red is a lipophilic fluorescent dye that can be used for CNPs containing abundant lipids, and its reliability has been widely verified [7].

**Figure 4:**
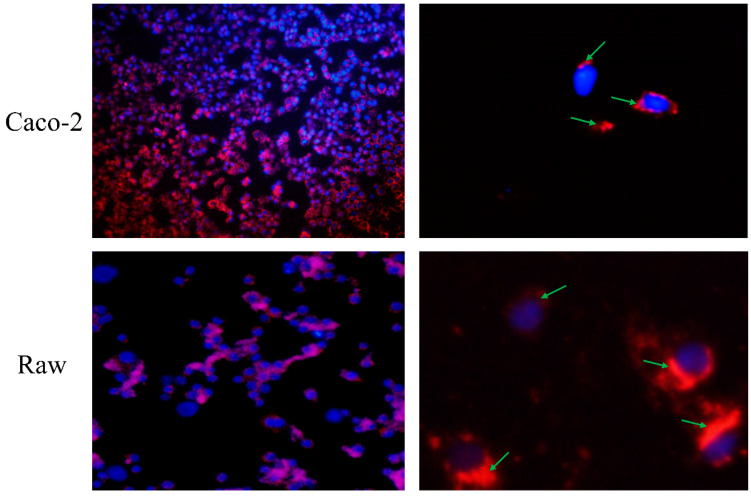
Observation of direct effects of Caco-2 cells and Raw 264.7 on the CNPs. The two images on the left are panoramic views, while the two images on the right are partial views. The nuclei were stained blue, while the CNPs were stained red. After incubation, the CNPs wrapped around the nucleus, as indicated by the green arrow.

As observed in Figure 4, after incubation, almost every cell nucleus was wrapped in the CNPs, and all regions of the cell except the nuclear region emitted a red fluorescence, indicating that the CNPs were not only attached to the cytoplasmic membrane, but also engulfed by the cells. The obtained CNPs from duck soup have been proven with significant antioxidant capacity, and here, their absorption by cells through the endocytic pathway implies the potential to improve the antioxidant capacities of cells.

### 3.5. Determination of Membrane Potential and Mitochondrial Superoxide Content in Cells

Oxidative stress is caused by an imbalance between reactive oxygen species (ROS) production and the antioxidant capacities of cells, which is a cellular state that is characterized by an excessive production of ROS [33,34]. ROS are produced by aerobic cells during metabolism, and the overproduction of ROS can cause cellular damage to intestinal epithelial cells [35,36]. Therefore, the body’s antioxidant system needs exogenous antioxidants to effectively avoid the occurrence of oxidative stress. According to several reports, the reduction in oxidative stress prevents intestinal barrier deterioration and lowers inflammatory reactions inside the gut [37,38,39]. AAPH is a free radical initiator that can release hydroxyl radicals upon the stimulation of cells, thus causing oxidative stress and some damage to cell membranes. High concentrations of AAPH can severely damage cells, causing oxidative stress and further activating uncoupling proteins on the mitochondria, leading to a decrease in mitochondrial respiration rate and thus reducing intracellular free radical levels [40,41].

**Figure 5:**
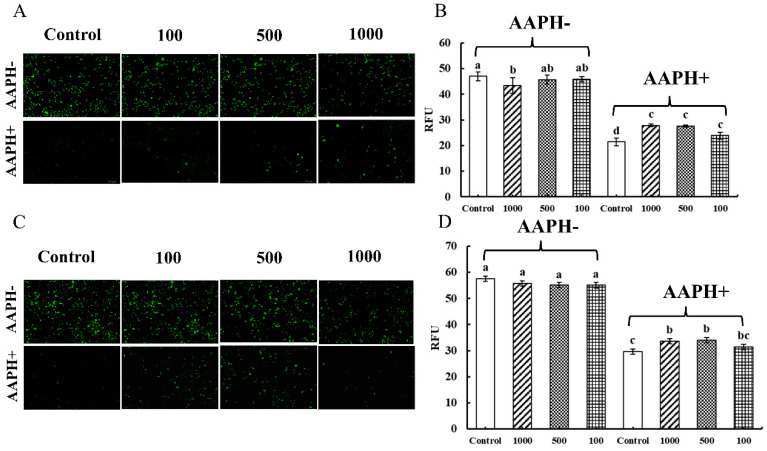
Determination of membrane potential in Caco-2 cells and Raw 264.7 cells. The effect of the CNPs at different concentrations on cell membrane potential in Caco-2 cells and Raw 264.7 cells are shown in (**A**) and (**B**), and in (**C**) and (**D**), respectively; different superscript letters indicate significant differences (*p* < 0.05).

In the absence of AAPH-induced cell damage (AAPH-), the fluorescence intensity of the groups in Caco-2 cells with additional CNPs was comparable to that of the control group, with no discernible differences based on the green fluorescence of DiBAC4(3) in Figure 5A. The relative fluorescence units (RFU) of Caco-2 cells were found to be much lower than those of the control group when the concentration of CNPs was 1000 µg/mL, as shown in Figure 5B. In Raw 264.7 cells, it was noticed that the fluorescence intensities of the groups with additional CNPs did not change substantially from that of the control group, based on the green fluorescence of DiBAC_4_(3) in Figure 5C. Moreover, the RFU of the groups added with various concentrations of CNPs did not differ noticeably from the control group in Figure 5D.

When the AAPH inducer was added to the cells, as shown in Figure 5 (AAPH+), the green fluorescence in Caco-2 cells and Raw 264.7 cells was extinguished, while a decrease in RFU could be observed. However, the fluorescence was significantly restored by the addition of CNPs, probably due to the alleviation of cellular damage caused by AAPH radicals, which significantly restored the cellular membrane potential and thus counteracted the hyperpolarized state of the cellular membrane caused by extracellular hydrogen peroxide radicals. For Caco-2 cells, the presence of AAPH has been reported to cause an increase in cell permeability, which can be reduced by CNPs [32]. CNPs can also protect the macrophage cytoplasm and membrane from AAPH-induced oxidative damage [20]. Therefore, it can be inferred that CNPs in the appropriate concentration range extracted from duck soup would rather protect than damage the membranes of Caco-2 and Raw 264.7 cells under oxidative stress.

The mitochondrion is the main site of ROS production in cells, and also the target organ of cellular oxidative stress damage [34]. Mito-Sox Red is a specific fluorescent indicator for the detection of reactive oxygen ROS levels, and its fluorescence intensity is proportional to the ROS concentration.

**Figure 6:**
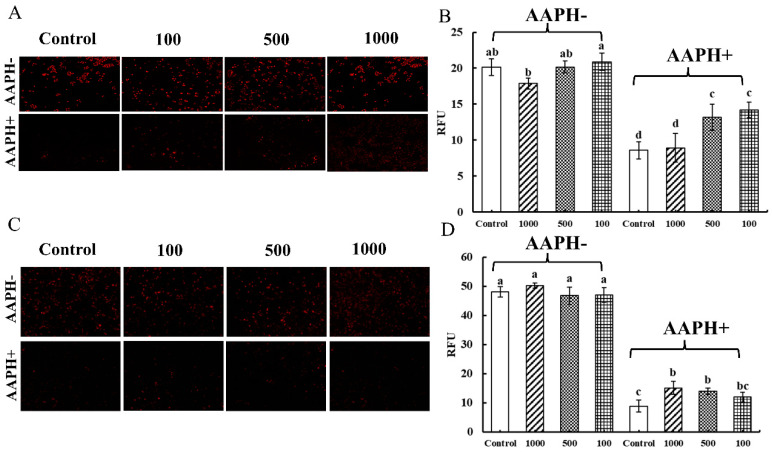
Determination of mitochondrial superoxide content in Caco-2 cells and Raw 264.7 cells. The effect of the CNPs at different concentrations on cell mitochondrial superoxide in Caco-2 cells and Raw 264.7 cells are shown in (**A**) and (**B**), and in (**C**) and (**D**), respectively; different superscript letters indicate significant differences (*p* < 0.05).

As shown in Figure 6A,C, there was no difference in the red fluorescence when Caco-2 cells and Raw 264.7 cells ingested the CNPs (AAPH-). As shown in Figure 6B,D, the fluorescence intensities of Caco-2 cells and Raw 264.7 cells were not significantly different from those of the control group, indicating that the CNPs in duck soup had almost no effect on mitochondrial reactive oxygen radicals. When the cells were subjected to AAPH radical-induced damage (AAPH+), as observed in Figure 6A,C, the fluorescence of Caco-2 cells and Raw 264.7 cells almost disappeared, and the strong fluorescence could hardly be seen under the microscope, which indicated that AAPH radicals could resist the oxygen respiration in mitochondria and the production of ROS. When CNPs were added to co-incubate with the cells, the red fluorescence in the cells was significantly restored compared to the control group, counteracting some of the inhibition of mitochondrial ROS by AAPH and increasing the production of ROS, indicating that CNPs could relieve the oxidative stress of cells. In Figure 6B, different concentrations of CNPs could restore the intracellular fluorescence intensity of Caco-2 cells compared to the control group, except for 1000 µg/mL of CNPs, which had no such effect. As shown in Figure 6D, compared with the AAPH group, different concentrations of CNPs significantly increased the intracellular fluorescence intensity of Raw 264.7 cells and promoted ROS proliferation in mitochondria. It was speculated that when the concentration of CNPs exceeds 1000 µg/mL, it might cause toxic damage to the cells, which in turn impaired the mitochondrial function, which was consistent with the detection of the effects of CNPs on the cell membrane potential. The experimental results showed that 100 µg/mL and 500 µg/mL CNPs could effectively maintain mitochondrial oxygen respiration and shield cells from the oxidative harm brought on by hydrogen peroxide radicals.

Interestingly, duck meat is considered to have a pyretolysis effect on the body in Chinese folk and Chinese medicine, and duck soup is highly popular [42]. Further investigations revealed that the consumption of duck meat reduced energy metabolism in rats [43]. In this study, CNPs extracted from duck soup benefited the growth of macrophages and intestinal epithelial cells, and had the effect of alleviating the oxidative stress of the cells, which has implications for explaining the potential antioxidant benefits of duck meat and soup. It is worth mentioning that further tests should be carried out in mice to validate the function of CNPs in duck soup.

## 4. Conclusions

In conclusion, this study successfully extracted bioactive colloidal nanoparticles from duck soup, verifying their antioxidant activity. In a suitable concentration range, the CNPs were able to interact directly with RAW 264.7 cells and Caco-2 cells, and alleviate their cellular damage when exposed to oxidative stress. This study will contribute to the extraction and application of food-derived CNPs for better efficacy, and promote new attempts at nanotechnology in the food field.

## Figures and Tables

**Table 1:** Colloidal properties of F_1_, F_2_, and F_3_.

	Hydrodynamic Diameter (nm)	Derived Count Rate (kcps)	PDI	ζ-Potential mV
F_1_	255.23 ± 12.77 ^a^	467.00 ± 29.4 ^a^	0.183 ± 0.017 ^c^	−4.35 ± 0.86 ^b^
F_2_	220.25 ± 7.45 ^b^	158.20 ± 1.30 ^b^	0.517 ± 0.011 ^a^	−5.15 ± 2.40 ^b^
F_3_	147.40 ± 17.14 ^c^	127.76 ± 7.92 ^b^	0.493 ± 0.002 ^b^	−11.44 ± 2.45 ^a^

The letters ^a^, ^b^, and ^c^ indicate significant differences (*p* < 0.05). PDI, polymer dispersity index.

**Table 2:** Major compositions of the CNPs.

CNPs	Lipids	Proteins	Carbohydrates
(µg/mL)	511.7 ± 41.8	307.9 ± 20.8	78.8 ± 5.9
%	51.2	30.8	7.9

%: Relative content of each component in the CNPs. CNPs, colloidal nanoparticles.

## Data Availability

Data used for all findings in this study are available upon request from the corresponding author.
